# The active metabolite of leflunomide, A77 1726, interferes with dendritic cell function

**DOI:** 10.1186/ar1727

**Published:** 2005-04-01

**Authors:** Bernhard M Kirsch, Maximilian Zeyda, Karl Stuhlmeier, Johannes Grisar, Josef S Smolen, Bruno Watschinger, Thomas M Stulnig, Walter H Hörl, Gerhard J Zlabinger, Marcus D Säemann

**Affiliations:** 1 Department of Internal Medicine III/Clinical Divisions of Nephrology and Dialysis, Medical University of Vienna, Vienna, Austria; 2 Department of Internal Medicine III/Clinical Divisions of Endocrinology and Metabolism, Medical University of Vienna, Vienna, Austria; 3 Ludwig Boltzmann Institute of Rheumatology, Vienna, Austria; 4 Department of Internal Medicine III/Clinical Division of Rheumatology, Medical University of Vienna, Vienna, Austria; 5 CeMM – Center of Molecular Medicine, Austrian Academy of Sciences, Vienna, Austria; 6 Institute of Immunology, Medical University of Vienna, Vienna, Austria

## Abstract

Leflunomide, a potent disease-modifying antirheumatic drug used in the treatment of rheumatoid arthritis (RA), exhibits anti-inflammatory, antiproliferative and immunosuppressive effects. Although most of the beneficial effects of leflunomide have been attributed to its antimetabolite activity, mainly in T cells, other targets accounting for its potency might still exist. Because of mounting evidence for a prominent role of dendritic cells (DCs) in the initiation and maintenance of the immune response in RA, we analyzed the effect of the active metabolite of leflunomide (A77 1726; LEF-M) on phenotype and function of human myleloid DCs at several stages in their life cycle. Importantly, DCs differentiated in the presence of LEF-M exhibited an altered phenotype, with largely reduced surface expression of the critical co-stimulatory molecules CD40 and CD80. Furthermore, treatment of DCs during the differentiation or maturation phase with LEF-M aborted successful DC maturation. Exogenous addition of uridine revealed that DC modulation by LEF-M was independent of its proposed ability as an antimetabolite. In addition, the ability of DCs to initiate T-cell proliferation and to produce the proinflammatory cytokines IL-12 and tumour necrosis factor-α was markedly impaired by LEF-M treatment. As a molecular mechanism, transactivation of nuclear factor-κB, an transcription factor essential for proper DC function, was completely suppressed in DCs treated with LEF-M. These data indicate that interference with several aspects of DC function could significantly contribute to the beneficial effects of leflunomide in inflammatory diseases, including RA.

## Introduction

Dendritic cells (DCs) are the most potent antigen-presenting cells in the immune system [1,2]. They represent a heterogeneous population of bone-marrow-derived cells located in lymphoid as well as in nonlymphoid organs. In peripheral tissues these antigen-presenting cells are immature and are functionally equipped to capture and process antigens. DCs are activated by pathogen-associated microbial patterns such as lipopolysaccharide (LPS) or by proinflammatory cytokines such as tumour necrosis factor (TNF)-α, and via the interaction of CD40 with its ligand (CD154), which is expressed on activated T cells [3]. Mature DCs possess optimal immunostimulatory properties because of maximal expression of their antigen-presenting and co-stimulatory molecules (i.e. CD40, CD80 and CD86) and their increased production of proinflammatory cytokines, including IL-12 and TNF-α. In contrast to the central role played by mature DCs in the initiation of primary immune responses, immature DCs stimulate T-cell responses only weakly or they may even induce tolerance to potential autoantigens [4].

Pharmacological modulation of DC activation has been demonstrated to prevent disease progression in several T-cell-mediated diseases [5], and it may therefore represent a promising approach to specific treatment of immunological disorders [6,7]. Notably, corticosteroids and another well known antirheumatic drug, namely gold thiomalate, significantly inhibit DC function, which may contribute to their clinical effectiveness [8,9].

Leflunomide is a novel disease-modifying antirheumatic drug that exerts its effects after metabolic opening of the isoxazole ring via its active metabolite A77 1726 (LEF-M). Its major target is supposed to be dihydro-orotate-dehydrogenase (DHODH) [10], which is a key enzyme in *de novo *pyrimidine synthesis. Leflunomide reversibly inhibits DHODH activity with subsequent depletion of nucleotides, leading to cell cycle arrest in proliferating lymphocytes [11]. This effect can be reversed to a certain degree by supplying the product of DHODH activity (i.e. uridine) to target cells. Other targets of LEF-M are tyrosine kinases such as Lck or JAK3 in activated T and B cells [12]. Immunosuppressive effects of leflunomide have been described including, inhibition of T cells and antibody production [13]. Furthermore, it was demonstrated that leflunomide blocks activation of nuclear factor-κB (NF-κB), which is a central proinflammatory transcription factor in several cell lines [14], and impairs transendothelial migration of peripheral blood mononuclear cells [15]. Apart from its well established beneficial effects in the treatment of rheumatoid arthritis (RA) [16,17], leflunomide is also effective in treatment against chronic allograft rejection [18,19].

DCs were postulated to play an important role in RA pathogenesis because they may perpetuate the disease by presenting self-antigen(s) [20,21]. Thus, DCs could represent an interesting target for dampening the disease process in RA. Moreover, DCs also play a fundamental role in allograft rejection [22].

Because the effect of leflunomide on DC function has not yet been investigated, we analyzed the influence of leflunomide on the complete DC life cycle *in vitro*. We found that LEF-M potently altered the phenotype and function of DCs, independent of its well known antimetabolite activity, revealing a novel immunomodulatory activity of this agent with potential clinical implications for the treatment of RA and other immune cell mediated disorders.

## Materials and methods

### Media and reagents

RPMI 1640 (GIBCO BRL, Grand Island, NY, USA) supplemented with 2 mmol/l L-glutamine, 100 μg/ml streptomycin, 100 U/ml penicillin and 10% foetal calf serum (FCS; Hyclone, Logan, UT, USA) was used as culture medium. LPS (*Escherichia coli *0111:B4) and uridine were purchased from Sigma Chemie GmbH Co. (Deisenhofen, Germany). Recombinant human (rh) granulocyte–macrophage colony-stimulating factor (GM-CSF) was obtained from Schering-Plough (Kenilworth, NJ, USA) and rh-IL-4 was from Strathmann Biotech GmbH (Hannover, Germany). Plasma concentrations in RA patients of A77 1726 (the active metabolite of leflunomide) achieved with a leflunomide maintenance dose of 20 mg/day are 46 ± 31 μg/ml (approximately 150 ± 100 μmol/l [23]). Therefore, we chose concentrations from 75 to 150 μmol/l of A77 1726 (kindly provided by Aventis, Strasbourg, France) for DC treatment. A77 1726 is referred to as 'LEF-M' throughout the report. In some experiments uridine was added to test the reversibility of the observed effects of LEF-M.

### Cell preparation and culture

Peripheral blood mononuclear cells were obtained from buffy coats of healthy blood donors (courtesy of the Austrian Red Cross) by density gradient centrifugation over Ficoll-Paque PLUS (Amersham Biosciences, Uppsala, Sweden). For isolation of monocytes, peripheral blood mononuclear cells were depleted of T cells by sheep erythrocyte-rosetting overnight.

Monocytes (>85% CD14^+^) were cultured in six-well plates (Costar, Cambridge, MA, USA) at a cell density of 5 × 10^5 ^cells/ml in RPMI 1640/10% FCS medium in a humidified atmosphere containing 5% carbon dioxide. For induction of DC differentiation, the culture medium was supplemented for 5 days with 50 ng/ml rh-GM-CSF and 10 ng/ml rh-IL-4. For initation of maturation, LPS (100 ng/ml) was added for an additional 48 hours. For the DC differentiation and maturation experiments, different concentrations of LEF-M, or medium as control, were added either at the beginning of the culture or 6 hours before the addition of LPS. Cell viability was assessed by staining with propidium iodide (PI; Sigma, Saint Louis, MO, USA) and subsequent flow cytometric analysis of the cells.

### Surface marker expression

For evaluation of surface marker expression, cells (50 μl at 5 × 10^6 ^cells/ml) were incubated with fluorescein isothiocyanate (FITC)-conjugated or phycoerythrin (PE)-conjugated mAbs for 45 min at 4°C. For control purposes, nonbinding isotype-matched FITC-conjugated and PE-conjugated mouse IgG (An der Grub, Kaumberg, Austria) were employed. After extensive washing cells were analyzed on a COULTER EPICS XL-MLC flowcytometer (Beckman Coulter, Fullerton, CA, USA) using EXPO32 software. All measurements were done using a three-colour setup, which was established using standard compensation procedures. FITC-labelled mAbs to CD1a (IgG_1_; clone HI149), CD14 (IgG_2b_; clone MΦP9), CD83 (IgG_1_; clone HB15e) and HLA-DR (IgG_2a_; L243), and R-PE-labelled mAbs to CD80 (IgG_1_; L307.4), CD86 (IgG_2b_; clone IT2.2) and CD206 (mannose receptor; IgG_1_; clone 19.2) were obtained from Becton Dickinson (San Diego, CA, USA). FITC-conjugated anti-CD40 (IgG_1_; clone LOB7/6) was purchased from Serotec (Oxford, UK). R-PE-labelled anti-major histocompatibility complex (MHC) class I antibody (IgG_2a_; clone 3F10) was obtained from Ancell (Bayport, MN, USA).

### Morphological cell analysis

Microscopy was performed in parallel to all other analyses to assess cell morphology by using a light optical microscope (Olympus Corporation, Tokyo, Japan).

### Assessment of T-cell stimulatory capability

Stimulator cells were irradiated (3000 rad, ^137^Cs source) and added at increasing cell numbers to 1 × 10^5 ^allogeneic T cells in 96-well culture plates in RPMI 1640 medium supplemented with 10% FCS (total volume 200 μl/well). After 4–5 days, cells were pulsed with 1 μCi [^3^H]thymidine (ICN Pharmaceuticals, Irvine, CA, USA). After another 18 hours the cells were harvested on glass-fibre filters (Packard, Meriden, CT, USA) and DNA-associated radioactivity was determined using a microplate scintillation counter (Packard, Meriden, CT, USA). DNA synthesis was expressed as mean counts/min of triplicate cultures.

### Measurement of cytokine production

DCs were differentiated and subsequently activated (100 ng/ml LPS) in the presence or absence of different concentrations of LEF-M. Cell-free supernatants were harvested 48 hours after cell activation. Cytokines were measured by sandwich enzyme-linked immunosorbent assays using matched pair antibodies. Capture as well as detection antibodies to human IL-12p40 were obtained from R&D Systems (Minneapolis, MN, USA). Antibodies to human TNF-α were from PharMingen (San Diego, CA, USA). Standards consisted of human recombinant material from R&D Systems. Assays were set up in duplicate and were performed in accordance with recommendations from the manufacturers. The lower limit of detection was 20 pg/ml for all cytokines.

### Analysis of nuclear factor-κB activation

NF-κB activation was assessed using an electrophoretic mobility shift assay (EMSA).

Nuclear extracts from DCs were prepared as described perviously [24]. Oligonucleotides resembling the consensus binding site for NF-κB (5'-AGTTGAGGGGACTTTCCCAGGC-3') and activator protein-1 (5'-CGCTTGATGACTCAGCCGGAA-3') were purchased from Santa Cruz Biotechnology (Santa Cruz, CA, USA). The double-stranded oligonucleotides used in all experiments were end-labelled using T4 polynucleotide kinase and [γ-^32^P]-ATP. After labelling, 5 μg nuclear extract was incubated with 120,000 counts/min labelled probe in the presence of 3 μg poly(dI-DCs) at room temperature for 30 min. This mixture was separated on a 6% polyacrylamide gel in Tris/glycine/EDTA buffer at pH 8.5. Control experiments were performed as described previously [25]. The specificity of NF-κB binding was proven using excess, unlabelled NF-κB probe that competed successfully for NF-κB binding, whereas an unrelated competitor (activator protein-1 oligonucleotide) did not (data not shown).

### Statistical analysis

Comparisons were performed using two-tailed paired Student's t-tests. *P *< 0.05 was considered statistically significant.

## Results

### LEF-M impairs differentiation of monocyte-derived dendritic cells

**Figure 1:**
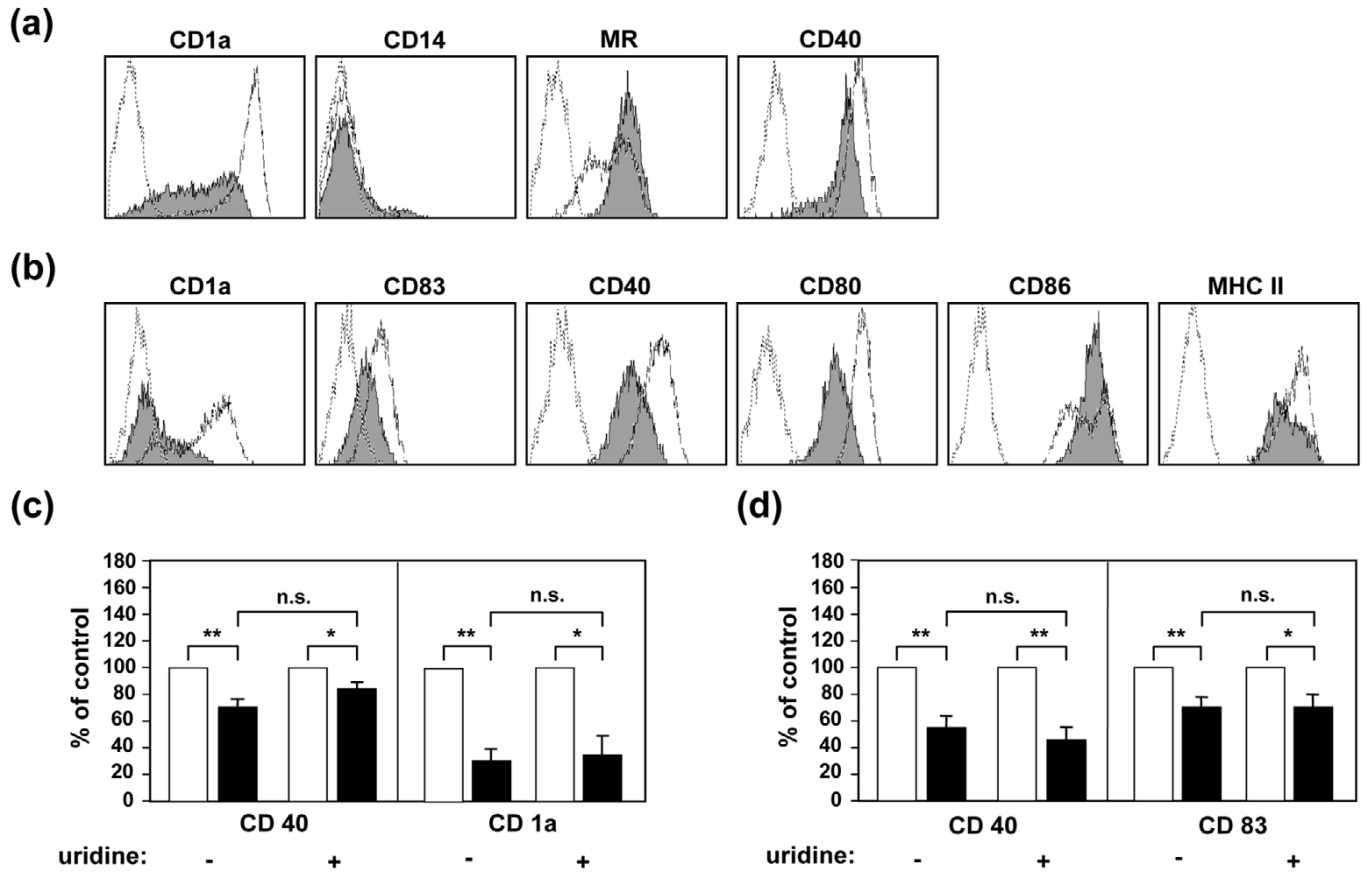
LEF-M interferes with DC differentiation. **(a) **Monocytes were cultured for 5 days with granulocyte–macrophage colony-stimulating factor (GM-CSF; 50 ng/ml) plus IL-4 (10 ng/ml) in the absence or presence of 150 μmol/l of the active metabolite of leflunomide (LEF-M). Subsequently, surface marker expression was determined using fluorescence-activated cell sorting (FACS) analysis. Open profiles with dotted line represent staining pattern with an isotype control antibody, open profiles with fine line indicate the staining pattern of differentiated control dendritic cells (DCs) stained with the indicated mAbs, whereas solid grey profiles show staining of DCs differentiated in the presence of LEF-M. **(b) **Myeloid precursor cells differentiated in the presence of LEF-M are resistant to maturation. Cells were treated as described above and then stimulated with lipopolysaccharide (LPS; 100 ng/ml) for 48 hours. Open profiles with dotted line represent staining pattern with an isotype control antibody, open profiles with fine line indicate staining of activated control DCs, and solid grey profiles show staining of DCs differentiated in the presence of LEF-M and subsequently exposed to LPS. Data are representative of at least four independent experiments. **(c,d) **The effects of LEF-M on DC differentiation are independent of pyrimidine depletion. The respective change in mean flourescence intensity (MFI) are shown **(c) **after the differentiation phase for CD40 and CD1a and **(d) **after subsequent maturation with 100 ng/ml LPS for CD40 and CD83 with and without 50 μmol/l uridine. White bars represent control DCs, and black bars indicate LEF-M-treated cells. Shown are mean percentage control responses ± standard error of the mean, calculated from five to eight independent experiments. Student's t-tests were calculated for control versus LEF-M-treated DCs and for LEF-M-treated DCs versus without uridine addition, as indicated. *P < 0.05, **P < 0.01.

In the first set of experiments we sought to determine whether leflunomide influences the differentation of freshly isolated monocytes into immature DCs. Therefore, we added GM-CSF and IL-4 to freshly isolated monocytes for 5 days to differentiate them to immature DCs in the presence or absence of LEF-M. Subsequently, we assessed surface marker expression using fluorescence-activated cell sorting analysis and found profound phenotypical differences between these differentiated cells. In the absence of LEF-M we found the typical immature DC phenotype, including high levels of MHC class II and high levels of CD1a, and a distinct profile of co-stimulatory molecules (Fig. 1a); neither the monocyte lineage marker CD14 nor the typical DC maturation marker CD83 was expressed. In contrast, LEF-M-treated DCs exhibited a different phenotype, with profoundly suppressed surface expression of CD40 and CD80 (Fig. 1a). Importantly, LEF-M markedly prevented the induction of the Langerhans cell-associated marker CD1a, which is a marker of successful DC differentiation, whereas expression of CD86, mannose receptor and MHC class I and II molecules remained unaffected by LEF-M (Fig. 1a). Of note, LEF-M did not interfere with the characteristic disappearance of the monocyte marker CD14. Furthermore, we found no difference in cell viability between LEF-M-treated and control cells, as determined by PI staining. As calculated from eight independent experiments, the percentage PI positivity was 13.8 ± 4.5% in untreated cells versus 14.3 ± 1.4% in cells treated with 150 μmol/l LEF-M (mean percentage ± standard error of the mean).

Addition of uridine did not rescue DC differentiation from the effects of LEF-M, indicating that inhibition of DHODH did not underlie the observed effects (Fig. 1c).

Finally, LEF-M-modulated DCs were assessed for maturation sensitivity. Although immature control DCs exposed to LPS exhibited typical features of mature DCs (Fig. 1b), including upregulation of CD40, CD80, CD86, MHC class I and II and neo-expression of CD83, the maturation program was arrested in cells that were differentiated and subsequently activated in the presence of LEF-M. As shown in Fig. 1b, LEF-M-treated DCs, despite LPS stimulation, continued to exhibit profoundly inhibited expression of CD40 and CD80, whereas LEF-M only marginally affected CD86 and MHC expression. Importantly, CD83 expression was abolished in LEF-M pretreated cells (Fig. 1b). Again, addition of uridine did not reverse the inhibitory effects of LEF-M on DC maturation (Fig. 1d). Again, the effects of LEF-M on DC phenotype were not simply a consequence of cellular cytotoxicity, as indicated by unchanged cell morphology and viability (percentage PI positivity was 9.0 ± 2.9% in untreated cells versus 15.3 ± 0.5% in cells treated with 150 μmol/l LEF-M; data expressed as mean percentage ± standard error of the mean, calculated from eight independent experiments).

### LEF-M impaires cytokine production and the allostimulatory capacity of monocyte-derived dendritic cells

**Figure 2:**
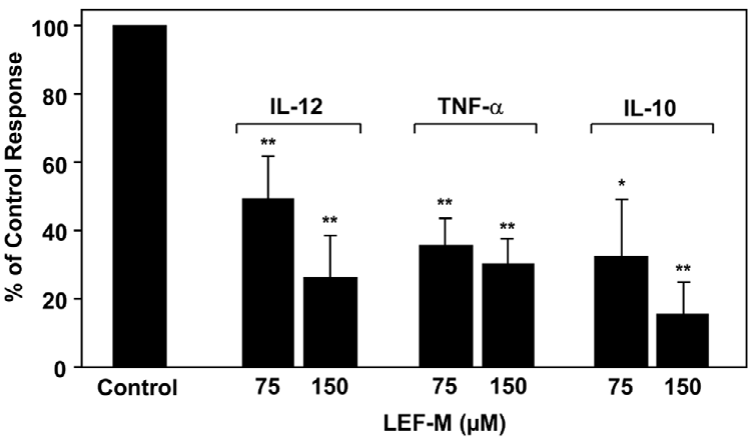
LEF-M abrogates cytokine production in DCs. Dendritic cells (DCs) were differentiated and subsequently activated (100 ng/ml lipopolysaccharide [LPS]) in the presence or absence of the indicated concentrations of the active metabolite of leflunomide (LEF-M). Cell-free supernatants were collected 48 hours after addition of LPS and then analyzed using enzyme-linked immunosorbent assay. Shown are mean percentage of control responses ± standard error of the mean for IL-12, tumour necrosis factor (TNF)-α and IL-10, calculated from at least 10 independent experiments. Student's t-tests were calculated for control DCs versus LEF-M-treated DCs. *P < 0.05, **P < 0.01. Mean cytokine levels (± standard deviation) in stimulated control cultures were 793 ± 343 pg/ml (IL-10), 23.6 ± 7.6 ng/ml (IL-12) and 2.9 ± 1.1 ng/ml (TNF-α).

DCs are typically characterized by their ability to produce large amounts of predominantly T-cell modulatory cytokines [26]. Analyzing cytokine production of cells that were differentiated and subsequently maturated in the presence of LEF-M, we found dose-dependent inhibition of IL-12p40 and TNF-α and of IL-10 production (Fig. 2).

**Figure 3:**
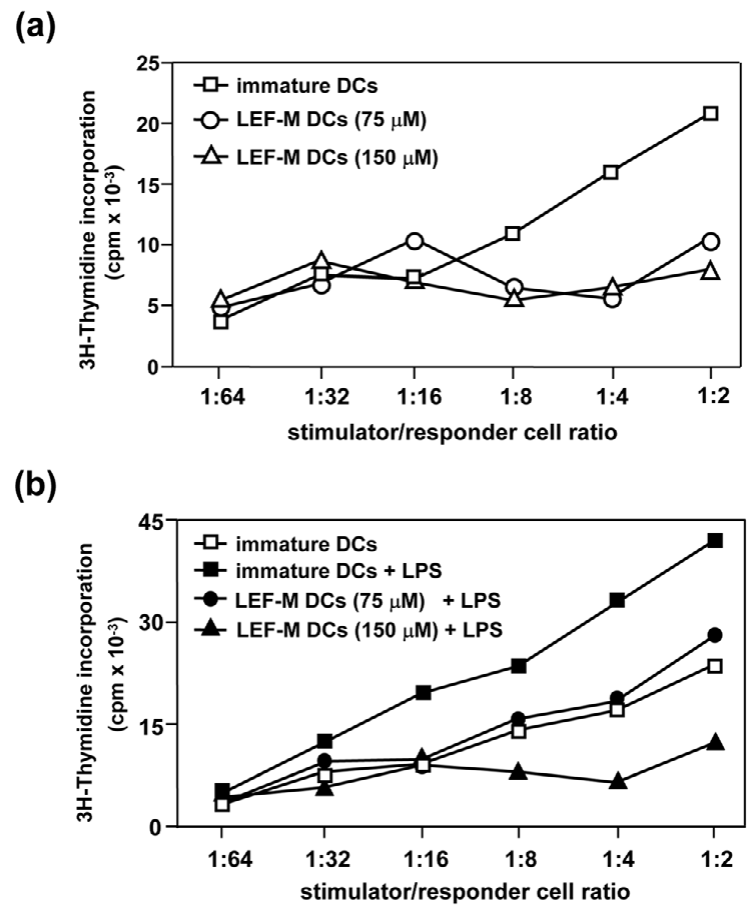
DCs differentiated in the presence of LEF-M exhibit reduced T-cell stimulatory capacity. **(a) **Monocytes were cultured for 5 days with granulocyte–macrophage colony-stimulating factor (GM-CSF; 50 ng/ml) plus IL-4 (10 ng/ml) in the presence or absence of the indicated concentrations of LEF-M. Dendritic cells (DCs) differentiated in the presence of the active metabolite of leflunomide (LEF-M) are labelled 'LEF-M DCs' in the figure. The cells were extensively washed, irradiated (3000 rad) and subsequently co-cultured with 1 × 10^5 ^purified allogeneic T cells at the indicated ratios. **(b) **To determine maturation sensitivity, DCs differentiated in the presence or absence of LEF-M were exposed to 100 ng/ml lipopolysaccharide for an additional 48 hours. Then, the cells were employed as allogeneic stimulators, as described above. DNA synthesis was assessed at day 5. The standard deviation of the counts/min (cpm) for the respective triplicates was generally below 20%. Shown are the means of at least eight independent experiments.

In addition to the observed distortion in DC phenotype after differentiation and maturation, we found profound impairment of the allo-stimulatory function of LEF-M pretreated DCs. As shown in Fig. 3a, immature control DCs exhibited poor stimulatory capacity of allogeneic T-cells. DCs differentiated in the presence of LEF-M were even less potent stimulators in the mixed leukocyte culture (Fig. 3a). LPS exposure dramatically increased the stimulatory capability of control DCs, but DCs differentiated in the presence of LEF-M and subsequently exposed to an activation stimulus were as ineffective as immature control DCs in supporting T-cell proliferation (Fig. 3b).

### LEF-M interferes directly with maturation of dendritic cells

**Figure 4:**
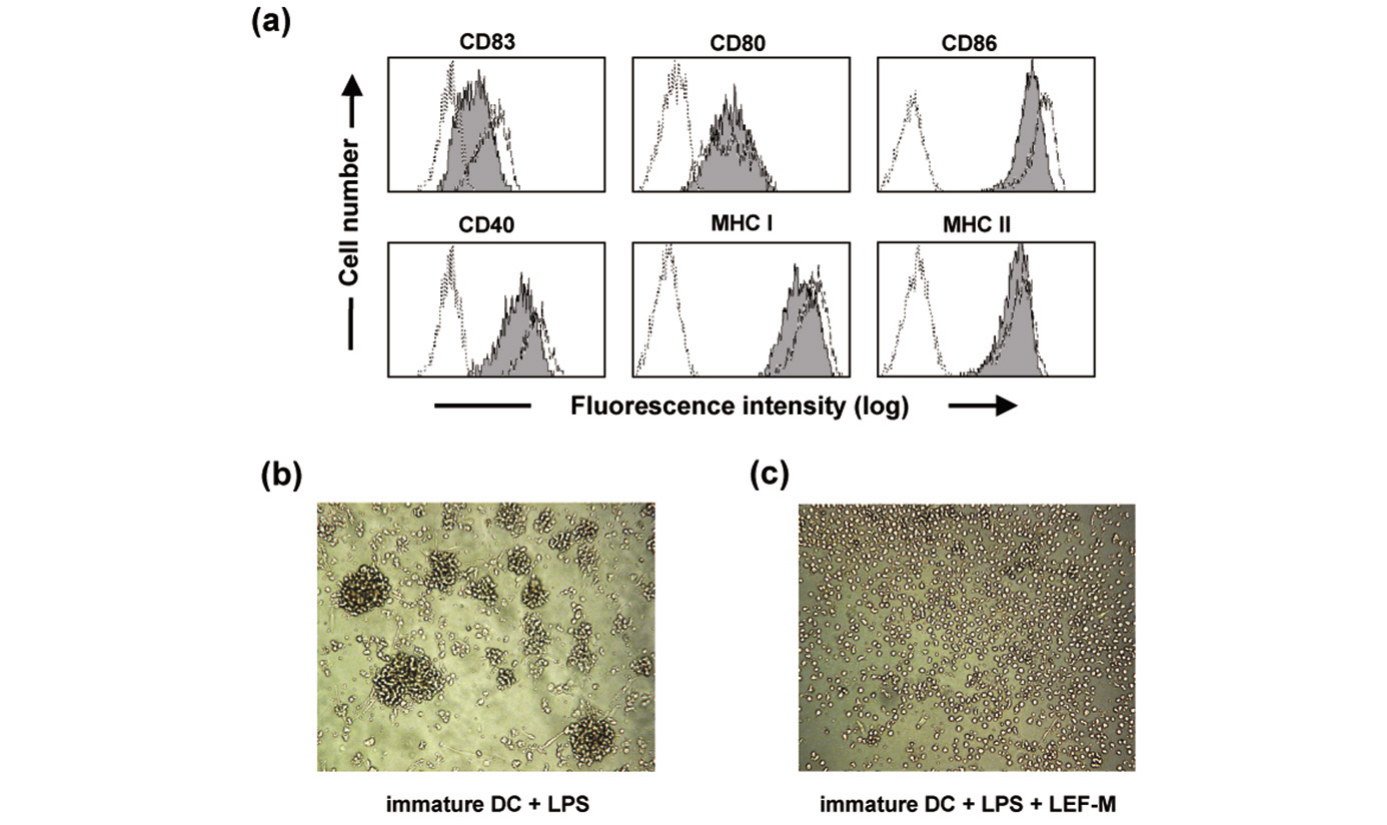
Treatment with LEF-M during maturation of immature DCs leads to a differentially affected phenotype. Monocytes were cultured for 5 days with granulocyte–macrophage colony-stimulating factor (GM-CSF; 50 ng/ml) plus IL-4 (10 ng/ml). **(a) **On day 5 these immature dendritic cells (DCs; 5 × 10^5^/ml) were activated with lipopolysaccharide (LPS; 100 ng/ml) in the absence or presence of 150 μmol/l of the active metabolite of leflunomide (LEF-M) for 48 hours. Surface marker expression was determined by fluorescence-activated cell sorting analysis. Open profiles with dotted line represent the staining pattern with an isotype control, open profiles with fine line indicate the staining pattern of DC exposed to LPS with the indicated monoclonal antibodies, and solid grey profiles show staining of DCs matured in the presence of LEF-M. The results shown are representative of five independent experiments. **(b,c) **Effect of LEF-M on maturation-associated clustering of DCs; immature DCs were stimulated with LPS in the (panel b) absence or (panel c) presence of 150 μmol/l LEF-M. After 8 hours of cultivation, cells were analyzed by inspecting photomicrographs obtained by light microscopy. Similar results were obtained in four additional experiments. MHC, major histocompatibility complex.

**Figure 5:**
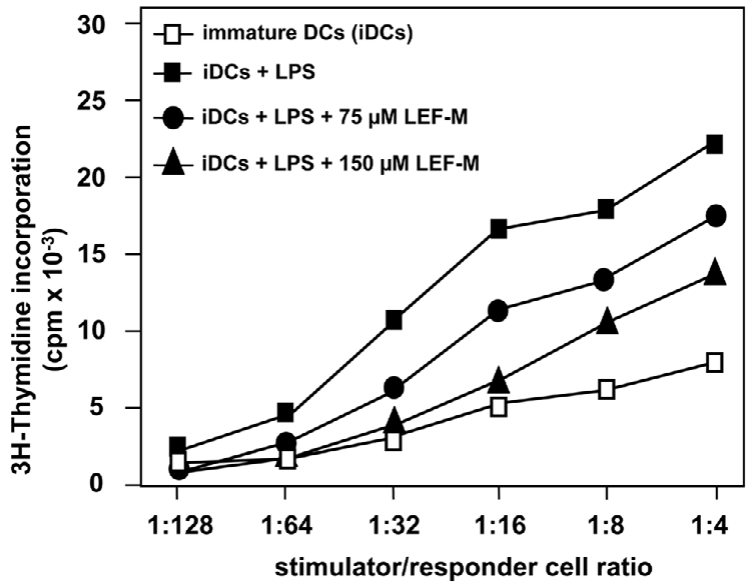
Functional impairment of DCs matured in the presence of LEF-M. Immature dendritic cells (iDCs) were exposed to lipopolysaccharide (LPS; 100 ng/ml) in the absence or presence of the indicated concentrations of the active metabolite of leflunomide (LEF-M). Then, the cells were extensively washed, irradiated (3000 rad) and subsequently co-cultured with 1 × 10^5 ^purified allogeneic T cells at the indicated ratios. DNA synthesis was assessed after 5 days and was measured in triplicate. The standard deviation of triplicates was generally below 20%. The data shown are expressed as mean counts/min (cpm) of four independent experiments.

We then analyzed whether LEF-M affects DC maturation when the drug was added to immature DCs (i.e. after completion of DC differentiation). Although immature control DCs responded readily, with increased expression of co-stimulatory and antigen-presenting molecules, LEF-M markedly interfered with the activation-induced upregulation of CD40 and CD86 but not that of CD80 (Fig. 4a). Importantly, neo-expression of CD83 – an indicator of proper DC maturation [27] – was significantly impaired in LEF-M-treated DCs (Fig. 4a). A further striking feature of mature DCs is the development of prominent cell clusters a few hours after addition of the maturation stimulus. On analyzing LEF-M-treated DCs, we detected complete abrogation of this clustering response (Fig. 4b,c). Another typical hallmark of mature DCs is their exceptional T-cell stimulatory capacity. As shown in Fig. 5, mature DCs exhibited optimal T-cell stimulatory capability. In contrast, the presence LEF-M solely during the maturation period of already differentiated DCs abrogated their stimulatory capacity in a concentration-dependent manner.

### Effect of LEF-M on nuclear factor-κB activation in dendritic cells

**Figure 6:**
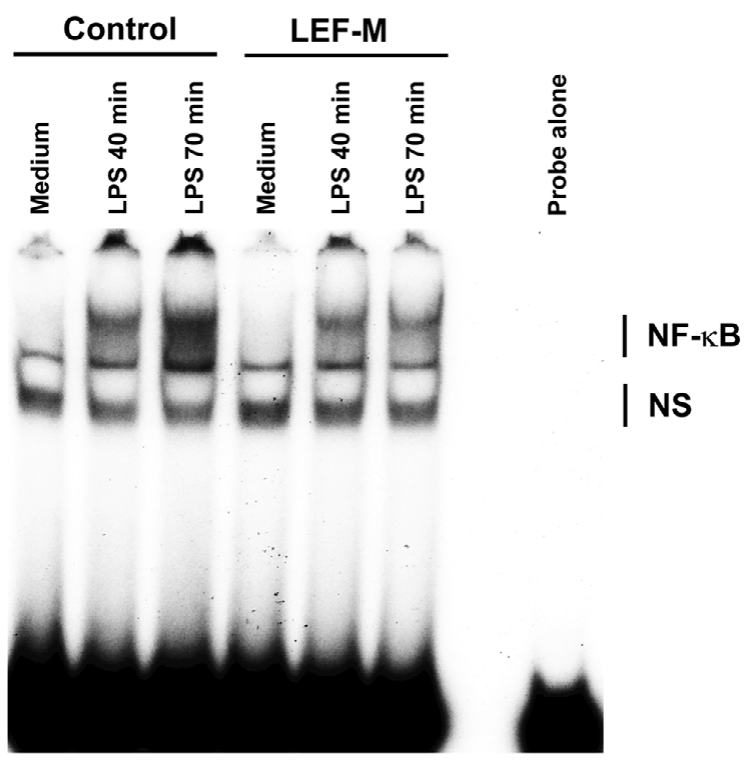
LEF-M suppresses LPS-induced NF-κB activation in DCs. Immature dendritic cells (DCs) were cultured for 2 hours with or without the active metabolite of leflunomide (LEF-M; 150 μmol/l), followed by addition of lipopolysaccharide (LPS; 100 ng/ml) or medium as control. After 40 and 70 min total nucleoprotein was extracted and nuclear factor-κB (NF-κB) activity was detected using electrophoretic mobility shoft assay. Similar results were obtained in two independent experiments. (Nonspecific bands are labelled NS.)

Activation of the transcription factor NF-κB is essential for DC function [28,29]. DCs readily respond to diverse stimuli such as microbial products, cytokines and tissue damage, all of which converge on the NF-κB pathway [30]. Our findings of an impaired DC function in LEF-M-treated cells prompted us to analyze the effect of LEF-M on the activation of this central transcription factor in DCs. As shown in Fig. 6, employment of electrophoretic mobility shift assays revealed a clear time-dependent increase in nuclear binding of the NF-κB consensus site upon LPS stimulation in DCs. The specificity of NF-κB binding was indicated by competition with unlabelled probe and an unrelated competitor (activator protein-1 oligonucleotide; data not shown). Strikingly, treatment of immature DCs with LEF-M profoundly suppressed nuclear translocation of NF-κB in LPS-stimulated DCs after both 40 and 70 min (Fig. 6).

## Discussion

This study reveals a novel aspect of the immunomodulatory action of leflunomide, namely the profound interference of LEF-M (A77 1726) with DC function. Using human monocyte-derived DCs as a model system, we demonstrated that LEF-M disrupts differentiation of DCs from uncommitted monocytic precursor cells, resulting in maturation-insensitive DCs. Furthermore, we showed that the maturation process of uncommitted immature DCs was markedly impaired by LEF-M. The metabolite LEF-M differentially affected the expression of critical surface molecules, inhibited the production of proinflammatory cytokines and, at the functional level, profoundly impaired the T-cell stimulatory capacity of DCs. As a molecular basis for the ability of LEF-M to interfere with several aspects of DC function, the activation-driven nuclear transmigration of the essential transcription factor NF-κB was markedly impaired by LEF-M. These findings have substantial implications for our understanding of the effects of leflunomide as a disease-modifying antirheumatic drug, because the initiation of an immune response critically depends on proper DC function. Furthermore, interference with DC maturation and function could also be involved in the beneficial effects of leflunomide on chronic allograft rejection [18], which is not shared by most other currently used immunosuppressive drugs such as calcineurin inhibitors.

The observation that DCs could play a pivotal role in the formation and maintenance of joint inflammation in RA [31] was confirmed by the finding reported by Balanescu and coworkers [32] of a correlation between co-stimulatory molecule expression of synovial DCs and disease activity in RA patients. Moreover, mature DCs might be central in the development of perivascular aggregates in synovial inflammation areas, the formation of organized lymphoid structures, and in the perpetuation of inflammatory and erosive activity [20,21]. Although there is sufficient evidence for an impact of leflunomide on synoviocytes, chondrocytes and osteoclasts [33-36], our data suggest that the potent inhibition of DC function by LEF-M might contribute to the beneficial effects of leflunomide treatment in patients with RA.

Exposure of DCs to LEF-M led to an alteration in the surface marker profile. Our findings concerning the impact of LEF-M on critical co-stimulatory molecules might be especially important in RA because the expression level of co-stimulatory molecules on DCs correlates with disease activity in patients with RA [32]. Another important finding in the present study was the observed disruption by LEF-M of the DC differentiation process. Interestingly, neo-expression of CD1a – the classic Langerhans cell-associated marker – was strongly inhibited in LEF-M-treated DCs. This finding is accordance with observations of significant efficacy of leflunomide in psoriasis [37], in which CD1a is highly overexpressed in involved skin [38]. Importantly, CD14 – a classic monocyte/macrophage marker – was downregulated, indicating that LEF-M does not subvert the DC differentiation programme toward macrophages as has been shown for IL-6, IL-10 and corticosteroids [39,40].

A central observation in our study was the functional alteration of DCs differentiated in the presence of LEF-M; these cells exhibited a marked reduction in their T-cell stimulatory capacity upon activation. These data indicate that LEF-M, by blocking the differentiation of monocytic precursors into mature DCs, potentially impairs proper DC function and might therefore modulate immune responsiveness against potential autoantigens and other antigens. Our finding of markedly decreased production of TNF-α and IL-12 by LEF-M-treated DCs, in conjunction with insufficient co-stimulatory molecule expression of DCs, may be of interest for further DC studies with LEF-M, because recent reports demonstrated this phenotype to be potentially tolerogenic [41,42].

Interestingly, we found the effects of LEF-M on DCs to be mediated independent of its inhibition of DHODH. As shown for several other leflunomide-mediated effects on other cell types, such as osteoclasts in the RA joint [43], memory T-cell lines in an autoimmune encephalomyelitis model [44] and in articular chondrocytes [34], or on functional effects such as repression of viral replication [45,46], the inhibitory effects of LEF-M in the present study are clearly independent of pyrimidine synthesis.

The transcription factor NF-κB plays a decisive role in proper DC function. NF-κB translocation is essential to the ability of mature DC to present antigen to naïve T cells [28,29]. Recently reported data demonstrate that LEF-M inhibits TNF-α-induced NF-κB activation in several cell lines [14,47]. Interestingly, we found a profound suppression of NF-κB transactivation in activated DCs by LEF-M. These results are in accordance with our findings showing impaired expression of maturation markers and reduced allo-stimulatory capacity of leflunomide-treated DCs, because selective inhibition of NF-κB activity has been shown to impair maturation of DCs [48]. Our findings concerning cytokine production are also consistent with NF-κB inhibition, because the human IL-12 promoter contains crucial NF-κB binding sites and TNF-α production is also NF-κB dependent [49]. Although the mechanisms underlying this profound NF-κB inhibitory activity of LEF-M on DCs are currently unknown, it is tempting to speculate that leflunomide may interfere with phosphorylation/dephosphorylation events in the LPS-triggered signalling program. Apart from the possibility that LEF-M might directly induce the transcription of distinct IκB family members, LEF-M could also induce particular phosphatases to inhibit the IκB-inactivating kinase IKK. Furthermore, recent studies have shown that leflunomide acts at the level of IκB-α phosphorylation via interference with IKK-α activation, ultimately leading to defective IκB-α phosphorylaton. Although further studies are required to unravel the detailed molecular mechanisms of suppressed NF-κB transactivation in LEF-M-treated DCs, our findings indicate that NF-κB inhibition is a central feature of the molecular actions of LEF-M on DCs.

Importantly, the results from the present study were obtained with monocyte-derived DCs generated from healthy volunteers. Hence, further studies will be necessary to clarify the effects of LEF-M on peripheral and synovial DCs in experimental models of arthritis and on DCs obtained from RA patients. Nevertheless, our finding of DC inhibition induced by LEF-M reveals a novel view of the disease-modifying effects of this drug, which appear to act on both T cells and DCs. In fact, the involvement of DC–T cell interactions in the pathways leading to and perpetuating RA and the effects of inhibiting this process are supported by recent findings on the significant clinical effects of interference with CD80/86–CD28 co-stimulation [50].

## Conclusion

The present study shows that monocyte-derived DCs are sensitive targets of LEF-M, possibly by inhibitory effects on NF-κB. DCs are affected by LEF-M at all major stages in their life cycle, ultimately leading to an impairment in DC function. In addition to a direct inhibitory action on specific T-cell responses, modulation of the immune system may therefore also be explained through the effects of leflunomide on DCs rendering these cells less able to support immunoinflammatory responses. Thus, the versatile role played by leflunomide as an immunomodulatory agent *in vitro *and *in vivo *is further supported by its effect on DCs. These findings reveal a novel mode of action of the active leflunomide metabolite during induction of cellular immune responses, which may contribute to the clinical effectiveness of leflunomide in diseases that involve exaggerated immune responsiveness.

## Abbreviations

DC = dendritic cell; DHODH = dihydro-orotate-dehydrogenase; FCS = fetal calf serum; FITC = fluorescein isothiocyanate; GM-CSF = granulocyte–macrophage colony-stimulating factor; IL = interleukin; LEF-M = active metabolite of leflunomide; LPS = lipopolysaccharide; mAb = monoclonal antibody; MHC = major histocompatibility complex; NF-κB = nuclear factor-κB; PE = phycoerythrin; PI = propidium iodide; RA = rheumatoid arthritis; rh = recombinant human; TNF = tumour necrosis factor.

## Authors' contributions

BK performed all flow cytometric and proliferation experiments, wrote the draft version of the manuscript and compiled the figures. MZ performed the uridine experiments. KS performed the electrophoretic mobility shift assays. JG analyzed the statistical data. JSS provided substantial input into the study design and helped in writing the manuscript. BW helped with statistical analysis and with finalizing the manuscript. WHH provided substantial input into the study design and helped with finalizing the manuscript. TMS was involved in all phases of the experimental process. GJZ performed the cytokine measurements. MDS designed the experiments, controlled all experimental steps and finalized the manuscript. All authors read and approved the final manuscipt.
